# Reversing Antibiotic Resistance Caused by Mobile Resistance Genes of High Fitness Cost

**DOI:** 10.1128/mSphere.00356-21

**Published:** 2021-06-23

**Authors:** Jinyong Wu, Xiaohong Dong, Lihua Zhang, Yufei Lin, Kun Yang

**Affiliations:** a Department of Pharmaceutical & Biological Engineering, School of Chemical Engineering, Sichuan University, Chengdu, China; JMI Laboratories

**Keywords:** antibiotic resistance genes, reversibility, fitness cost, qPCR, multidrug resistance, *mcr-1*

## Abstract

The reversibility of antibiotic resistance is theoretically attractive due to the prospect of restoring the clinical potency of antibiotics. It is important to find out the factors that affect the reversibility of antibiotic resistance. Here, an *mcr-1*-positive multidrug-resistant (MDR) environmental Escherichia coli isolate was successively passaged under four antibiotic-free culture conditions. The relative abundances of multiple antibiotic resistance genes (ARGs) kept decreasing during the successive passages. The linear correlations between abundances of ARGs on the same MDR plasmid reflected that the decay of antibiotic resistance during the passage was mainly due to the elimination of the MDR plasmid (pMCR_W5-6). Colistin-susceptible strains were isolated at the end of the passage. The whole-genome sequencing of two susceptible isolates detected the elimination of the MDR plasmid and deletion of the *mcr-1* gene. Deletions of DNA fragments from chromosome and plasmid were closely related to a variety of insertion sequences (ISs). The results of coculture of resistant and susceptible strains at different antibiotic concentrations indicated that the high fitness cost led to the poor stability of mobile ARGs. Strict control of the use of antibiotics can at least reverse the severe antibiotic resistance caused by mobile ARGs of high fitness cost.

**IMPORTANCE** The dissemination of bacterial antibiotic resistance is a serious threat to human health. The development of new antibiotics faces both economic and technological challenges. The reversibility of antibiotic resistance has become an important issue causing wide concern due to the prospect of restoring the clinical potency of antibiotics. Our study suggests that the high mobility of ARGs of high fitness cost may just reflect their poor stability. Therefore, strict control of the use of antibiotics can at least reverse the severe antibiotic resistance caused by mobile ARGs of high fitness cost. This study brings hope for the possibility of curbing the dissemination of antibiotic resistance.

## INTRODUCTION

The widespread use and even abuse of antibiotics in clinic, animal husbandry, and aquaculture induced (enriched) a large amount of antibiotic resistance (resistant bacteria, resistant plasmids, and resistance genes) (1). The dissemination of antibiotic resistance is closely related to the horizontal transfer of antibiotic resistance genes (ARGs), which is in response to antibiotic selection (2). The development of new antibiotics faces both economic and technological challenges (3). The reversibility of antibiotic resistance, which determines whether the existing antibiotics can restore their clinical potency, has become an important issue causing wide concern (4). Studies have shown that exposure to antibiotics or specific environments can rapidly increase the level of bacterial antibiotic resistance in individuals (5, 6). But with the relaxation of antibiotic selective pressure, the eradication of antibiotic resistance in bacterial populations is not straightforward, especially at the community level (4, 7). The antibiotic resistance was only partially reversed over a significant period of time after retreating from corresponding antibiotic or environmental effects (5, 6). The fitness cost of antibiotic resistance is the main biological factor that influences its reversibility. High fitness cost will allow susceptible bacteria to outcompete resistant ones without selective pressure from corresponding antibiotics (4). Most ARGs are located on plasmids and can be transferred between different bacterial strains or even between different bacterial species via conjugation (8). The fitness cost of a plasmid mainly comes from its metabolic burden, the interaction between the plasmid and the bacterial host, and the possible toxicity derived from the plasmid (9, 10). The presence of resistance genes on plasmids also tends to increase the fitness cost of the host bacteria (11, 12). Therefore, it is essential to determine the main source of bacterial fitness costs. Additionally, the different resistance mechanisms of resistance genes will also affect the fitness of resistant bacteria under antibiotic selective pressure in the bacterial community (13). Theoretically, resistance mechanisms such as efflux pumps, reduced membrane permeability, and antibiotic target alteration are only beneficial to the resistance gene-carrying bacteria, which will enable resistant bacteria to outcompete susceptible ones at a very low antibiotic concentration (14, 15). In contrast, resistance mechanisms like β-lactamases, inactivating the antibiotic via hydrolysis, will benefit the growth of surrounding bacteria, and resistant bacteria only show advantages under high enough selective pressure (16). It is very important to determine the minimum antibiotic concentration at which drug-resistant bacteria show competitive advantage for the formulation of antibiotic discharge standards.

For the bacterial antibiotic resistance caused by mobile ARGs, the reversibility of antibiotic resistance may arise from the deletion of individual ARGs or the elimination of the whole resistant plasmid. It is not clear which scenario is dominant as well as what kind of DNA sequence structure is more likely to lead to the loss of ARGs. So far, the stabilities of mobile ARGs or resistant plasmids and their fitness cost are rarely studied at the DNA molecular level. In this present study, the reversibility of antibiotic resistance was verified through multiple experimental designs. An *mcr-1*-positive multidrug-resistant Escherichia coli strain was successively passaged to detect the stability of multiple ARGs. The linear correlation analysis of abundances between different ARGs reflected the decay mode of antibiotic resistance, the deletion of individual ARGs, or the elimination of the whole MDR plasmid. Two colistin-susceptible strains isolated at the end of the passage were subjected to whole-genome sequencing to determine the loss mode of ARGs. The fitness cost of ARGs was evaluated by coculturing the resistant and susceptible strains at different antibiotic concentrations.

## RESULTS AND DISCUSSION

### Decay of ARGs.

**FIG 1:**
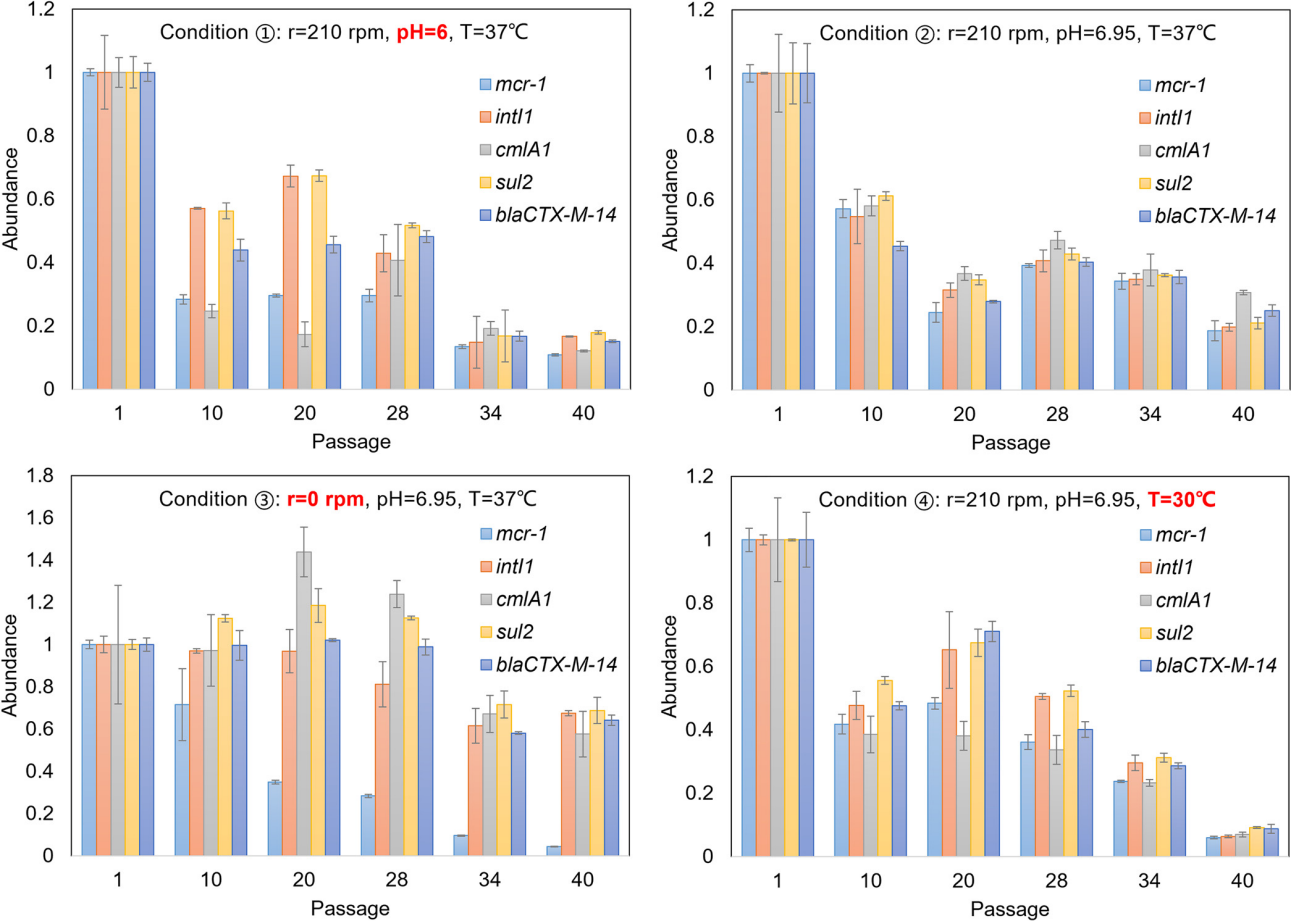
qPCR-determined and normalized relative abundances of typical ARGs (including *intI1*) during the successive passages at four antibiotic-free cultural conditions. Overall, the abundances of ARGs kept decreasing during successive passages. Static culture without shaking (condition 3) facilitated the maintenance of multiple ARGs, while the loss of *mcr-1* became the worst at this condition.

Although the abundances of ARGs kept decreasing during the successive passage under all four conditions and even decreased by more than 90% under condition 4 (the condition with lower incubation temperature; Fig. 1), the ARGs remained at low but detectable levels at the end of the passage, which is consistent with previous reports (7). The static culture without shaking seemed to be beneficial to the maintenance of the MDR plasmid, while the loss of *mcr-1* at this culture condition was the worst (Fig. 1, condition 3). Since these ARGs (including *intI1*) are on the same MDR plasmid, the linear correlation between their relative abundances can reflect the mode of ARGs’ decay, either the elimination of the whole MDR plasmid or the deletion of individual ARGs. The Pearson correlation coefficients between relative abundances of different ARGs were calculated and are listed in Table 1. In most cases, the linear correlations with high significance were observed, which meant that the decay of antibiotic resistance during the successive passage was mainly caused by the elimination of the whole MDR plasmid (pMCR_W5-6). The correlation between relative abundances of certain ARGs (such as *mcr-1* or *cmlA1*) and other ARGs under typical culture conditions deviated from linearity, especially *mcr-1* under culture condition 3 (i.e., the static culture) or *cmlA1* under condition 2 with lower pH (Table 1), which suggested that individual ARG (*mcr-1* or *cmlA1*) was deleted from the MDR plasmid (Fig. 1). Therefore, environmental factors may affect the persistence of individual ARGs on the plasmid and thus the stability of plasmid, which deserves more in-depth research. However, we also have noticed that different resistance genes are assembled with/into different mobile genetic elements. The deletion of *mcr-1* off the plasmid should be related to the insertion sequence IS*Apl1* (17), while for *cmlA1*, as a cassette gene of the integron In640, its excision relies on the activity of the *intI1*-encoded integrase (18).

**TABLE 1:** Pearson correlation coefficients between qPCR-determined relative abundances of different ARGs^*d*^

Gene	Pearson correlation coefficient for genes under:																			
	Condition 1^*c*^					Condition 2^*c*^					Condition 3^*c*^					Condition 4^*c*^				
	*mcr-1*	*cmlA1*	*intl1*	*sul2*	*bla*_CTX-M-14_	*mcr-1*	*cmlA1*	*intl1*	*sul2*	*bla*_CTX-M-14_	*mcr-1*	*cmlA1*	*intl1*	*sul2*	*bla*_CTX-M-14_	*mcr-1*	*cmlA1*	*intl1*	*sul2*	*bla*_CTX-M-14_
*mcr-1*	1	0.970^*b*^	0.887^*a*^	0.889^*a*^	0.970^*b*^	1	0.993^*b*^	0.995^*b*^	0.994^*b*^	0.979^*b*^	1	0.317	0.845^*a*^	0.528	0.703	1	0.992^*b*^	0.976^*b*^	0.971^*b*^	0.968^*b*^
*cmlA1*	0.970^*b*^	1	0.776	0.797	0.931^*b*^	0.993^*b*^	1	0.996^*b*^	0.991^*b*^	0.989^*b*^	0.317	1	0.699	0.923^*b*^	0.852^*a*^	0.992^*b*^	1	0.944^*b*^	0.937^*b*^	0.929^*b*^
*intl1*	0.887^*a*^	0.776	1	0.994^*b*^	0.948^*b*^	0.995^*b*^	0.996^*b*^	1	0.997^*b*^	0.985^*b*^	0.845^*a*^	0.699	1	0.837^*a*^	0.928^*b*^	0.976^*b*^	0.944^*b*^	1	0.997^*b*^	0.986^*b*^
*sul2*	0.889^*a*^	0.797	0.994^*b*^	1	0.962^*b*^	0.994^*b*^	0.991^*b*^	0.997^*b*^	1	0.969^*b*^	0.528	0.923^*b*^	0.837^*a*^	1	0.957^*b*^	0.971^*b*^	0.937^*b*^	0.997^*b*^	1	0.984^*b*^
*bla*_CTX-M-14_	0.970^*b*^	0.931^*b*^	0.948^*b*^	0.962^*b*^	1	0.979^*b*^	0.989^*b*^	0.985^*b*^	0.969^*b*^	1	0.703	0.852^*a*^	0.928^*b*^	0.957^*b*^	1	0.968^*b*^	0.929^*b*^	0.986^*b*^	0.984^*b*^	1

a Significance level at *P* value of <0.05 (*n* = 6, two-tailed test).

b Significance level at *P* value of <0.01 (*n* = 6, two-tailed test).

c Condition 1, *r* = 210 rpm, pH 6, 37°C; condition 2, *r* = 210 rpm, pH 6.95, 37°C; condition 3, *r* = 0 rpm, pH 6.95, 37°C; condition 4,  *r* = 210 rpm, pH 6.95, 30°C.

d Shading represents ignificant linear correlations.

### Deletion of ARG and elimination of the MDR plasmid.

**FIG 2:**
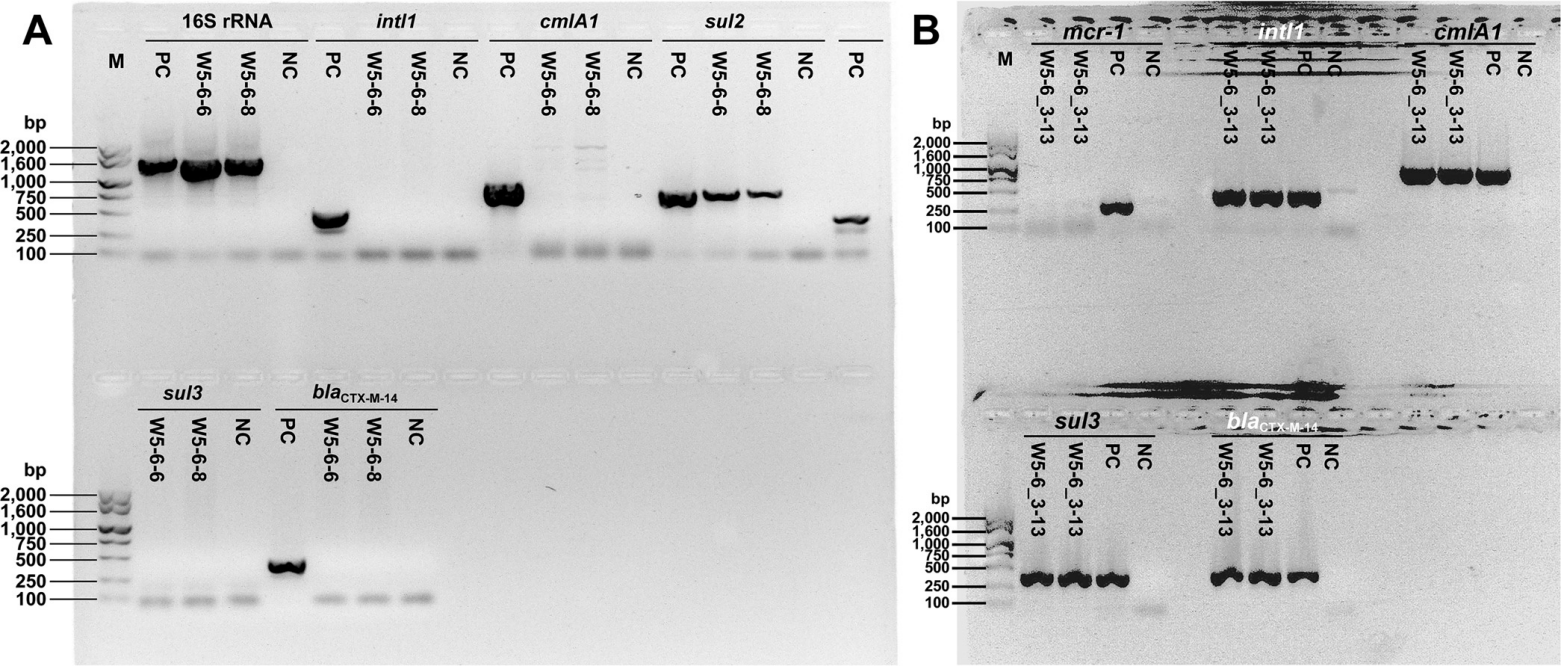
PCR detecting the presence of multiple ARGs in typical colistin-susceptible strains isolated at the end of the successive passage. E. coli W5-6-6 and W5-6-8 were isolated from cultural condition 2, i.e., the normal condition for liquid-phase culture of E. coli in our lab. Both isolates lost the *mcr-1* gene and other ARGs on the MDR plasmid pMCR_W5-6. (A) The *sul2* gene was also present on the chromosome of the original E. coli W5-6, so it was still detected but with relatively low abundance. E. coli W5-6_3-13 was isolated from cultural condition 3, i.e., the static culture. (B) The isolate lost the *mcr-1* gene but kept all other ARGs on the MDR plasmid. PC, positive control with DNA extract from original E. coli W5-6 as the template; NC, negative control.

**FIG 3:**
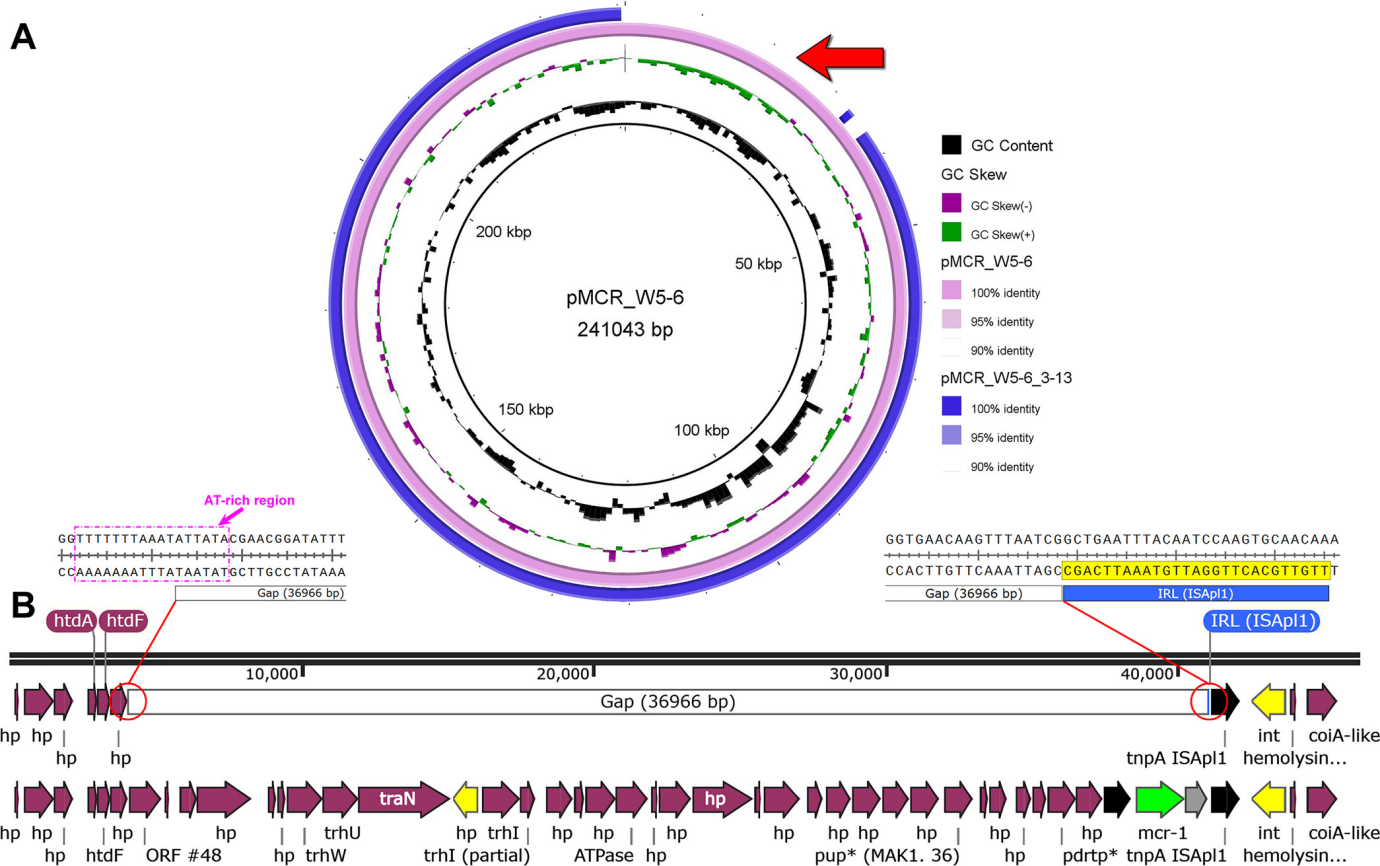
(A) Sequence alignment of the MDR plasmid in the original E. coli W5-6 and E. coli W5-6_3-13. The alignment was performed using BRIG tool (35). (B) Deletion of the DNA fragment on the plasmid pMCR_W5-6_3-13 is indicated with a red arrow and illustrated in detail. The right side of the missing DNA fragment (the gap) is closely next to the end of IS*Apl1*, while its left side is in an AT-rich region, which is the target hot spot of IS*Apl1*. IRL means the inverted repeat at the left end of the IS.

Colistin-susceptible E. coli strains were isolated at the end of the successive passage. According to the results of PCR, in addition to the loss of *mcr-1* gene in all these isolates, most of them (13 out 14 isolates) also lost other ARGs (including *intI1*) (Fig. S1 in the supplemental material), which meant that the MDR plasmid had been most likely eliminated from these isolates. The only exception is the isolate E. coli W5-6_3-13. According to the PCR results (Fig. 2B), it lost the *mcr-1* gene but retained other ARGs and *intI1*. Interestingly, E. coli W5-6_3-13 was just isolated from culture condition 3, where the correlation between relative abundances of *mcr-1* and other ARGs deviated from linearity (Table 1), which implied the deletion of the *mcr-1* gene from the MDR plasmid. The two representative E. coli isolates, W5-6-8 and W5-6_3-13, were subjected to whole-genome sequencing. The primary genomic information of the two isolates is also listed in Table 2. E. coli isolates W5-6-6 and W5-6-8 were isolated from condition 2 and lost almost all ARGs on the MDR plasmid (Fig. 2A). Therefore, we speculated that they eliminated the whole MDR plasmid. The *sul2* gene also presented on the chromosome of the original E. coli W5-6 (17), so it was still detected in colistin-susceptible E. coli isolates W5-6-6 and W5-6-8 and showed a relatively lower abundance in E. coli W5-6-8 (Fig. 2A). Whole-genome sequencing showed that W5-6-8 eliminated the MDR plasmid pMCR_W5-6 (Table 2). Although E. coli W5-6_3-13 kept the MDR plasmid, the plasmid had been deleted a *mcr-1*-containing DNA fragment of around 37,000 bp (Table 2 and Fig. 3). Recently, similar plasmid elimination and *mcr-1* deletion have been observed for *mcr-1*-bearing plasmids of other Inc types via nanopore sequencing (19, 20), which proves the universality of these phenomena. We named the truncated plasmid pMCR_W5-6_3-13 to distinguish it from the original form. Detailed sequence alignment showed that the right end of the deleted fragment was exactly broken next to the IS*Apl1*, while the left end was in an AT-rich region (Fig. 3B), which was previously reported to be the hot spot for the insertion of IS*Apl1* (17). Therefore, another IS*Apl1* should have been inserted into the AT-rich region and mediated the fragment deletion off the plasmid (20). The missing DNA fragment also contains other functional genes, such as the genes related to plasmid conjugation (*htdF*, *trhW*, *trhU*, and *trhI*). Based on the existing data and information, we can hardly judge whether the loss of these genes is directly or indirectly related to the fitness evolution of the drug-resistant bacterium, so we keep this information for future researchers to refer to. For the same reason, we also keep the primary annotations for the missing fragment on the chromosome in the next section.

**TABLE 2:** Genome composition in the original multidrug-resistant E. coli W5-6 and two colistin-susceptible isolates

Strain	No. of bp in:			
	Chromosome, ST2^*a*^	pMCR_W5-6, IncHI2^*a*^	p2_W5-6, IncX1^*a*^	p3_W5-6, IncN^*a*^
W5-6	4,638,901 (GenBank accession no. CP032992)	241,043 (GenBank accession no. CP032993)	44,779 (GenBank accession no. CP032994)	72,717 (GenBank accession no. CP032995)
W5-6-8	4,579,340 (−59,561)^*b*^	ND^*c*^	44,774 (−5)^*b*^	72,724 (+7)^*b*^
W5-6_3-13	4,635,411 (−3,490)^*b*^	204,074 (−36,969)^*b*^	44,774 (−5)^*b*^	72,668 (−49)^*b*^

a Strain type (ST) or incompatibility replicon type of plasmid (Inc).

b Numbers in brackets indicate the size change of the corresponding contig in comparison to the original strain (E. coli W5-6).

c ND, not detected.

10.1128/mSphere.00356-21.1 FIG S1 PCR detecting the presence/absence of ARGs in colistin-susceptible strains isolated from the last generation. Negative control for the *mcr-1* should be contaminated. Two representative isolates subjected to whole-genome sequencing are labeled in yellow and red, respectively. Since the culture condition 2 is the normal condition for liquid-phase culture of E. coli in our lab, we abbreviated the names of the strains isolated from this condition by omitting the number 2 that indicated the culture condition, i.e., W5-6-8 in the context was exactly W5-6_2-8 herein. PC, positive control with DNA extract from original E. coli W5-6 as the template; NC, negative control. Download FIG S1, JPG file, 2.1 MB. Copyright © 2021 Wu et al. 2021 Wu et al. https://creativecommons.org/licenses/by/4.0/ This content is distributed under the terms of the Creative Commons Attribution 4.0 International license.

In the process of bacterial cell division, the MDR plasmid might be distributed unevenly in the daughter cells. The MDR plasmid-negative clone would propagate faster than the positive one during the successive passage due to the lower fitness cost. However, static culture facilitated the plasmid conjugation between bacterial cells (21), which might couple with the process of *mcr-1* deletion. The deletion of the *mcr-1* gene compensated for its fitness cost caused by the modification of the structure of membranal lipid A (22). Under this condition, the relative better stability of the other ARGs (compared to the other three conditions) indicated that the MDR plasmid with *mcr-1* deletion was better maintained in the host bacteria, and its content was significantly higher than that of the *mcr-1*-positive one in the culture. The propagation of the *mcr-1*-negative MDR plasmid under culture condition 3 might arise from its comparative advantage in fitness over the *mcr-1*-positive one. As a result, the decrease of other ARGs was alleviated to some extent, while the decay of *mcr-1* became the worst at this condition. Such a special phenomenon suggests that the fitness cost of the MDR plasmid may mainly come from the *mcr-1* gene. The decay of the MDR plasmid in the bacterial population can be due to the restricted use of special antibiotics with corresponding ARGs of high fitness cost, which is just like the inverse process of coselection among ARGs when antibiotic resistance blooms under high antibiotic selective pressure.

In this work, we only detected the elimination of the MDR plasmid of the biggest size. The other two plasmids of smaller size were still in the two sequenced E. coli isolates, and there was no significant change in their sequences (Table 2). It is difficult to draw a conclusion about the relationship between plasmid size and stability because of the small number of samples we studied. Although in principle, large plasmids contain more coding sequences and their expression should consume more cell resources, so their fitness cost should be higher, it has been proven that the plasmid size does not correlate with its fitness cost or conferring multidrug resistance (23). Our result indicates that the fitness cost of an MDR plasmid may be dominated by individual ARGs. Although we did not observe the elimination of the other two small plasmids (Table 2), we noticed the presence of ARGs on these two small plasmids, such as *bla*_TEM−1_, *floR*, and *vgaC* (refer to our previous study [24]). Whether the fitness cost of these resistance genes will also lead to the elimination of plasmids or the deletion of individual ARGs is worthy of further study.

### Deletion of DNA fragments from the chromosome.

**FIG 4:**
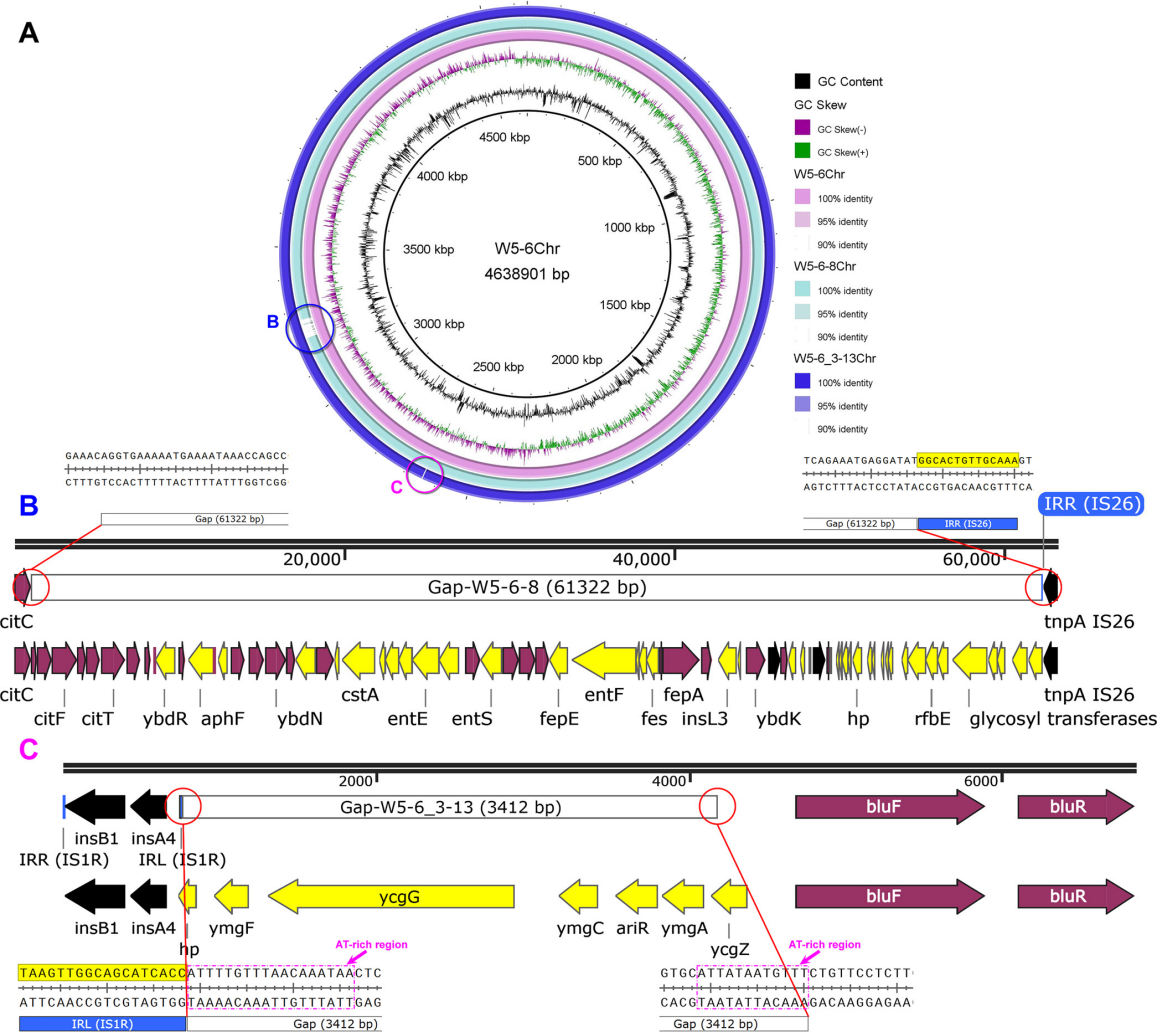
(A) Sequence alignment of the chromosomes of the original E. coli W5-6 and the two colistin-susceptible isolates. (B and C) Deletion of DNA fragments on W5-6-8Chr and W5-6_3-13Chr are indicated with blue (B) and magenta (C) circles. Both missing DNA fragments are closely next to the end of an IS (IS*26* and IS*1R*, respectively). Both ends of the missing DNA fragment on the chromosome W5-6_3-13Chr (i.e., Gap-W5-6_3-13) are in AT-rich regions which are the target hot spots of IS*1R*. IRL and IRR, inverted repeats on the left and right ends of ISs, respectively.

Besides the deletion of DNA fragments on the MDR plasmid, we also observed the deletion of DNA fragments from chromosome. Compared with the original E. coli W5-6, the two susceptible isolates each lost one segment of DNA from their chromosomes, respectively (Fig. 4A). The length of the missing fragment from the chromosome of E. coli W5-6-8 is 61,322 bp (Fig. 4B), and that of E. coli W5-6_3-13 is much smaller, only 3,412 bp (Fig. 4C). We found that the deletions of these DNA fragments from chromosome were like that on the plasmid and showed the same characteristic. The breakpoint of the deleted fragment is closely adjacent to an IS. For E. coli W5-6-8, the IS at the breakpoint is IS*26* (Fig. 4B). For E. coli W5-6_3-13, it is IS*1R* (an IS of IS1 family; Fig. 4C). Similarly, both ends of the missing DNA fragment on chromosome W5-6_3-13Chr are in AT-rich regions. The insertion diagrams in Fig. 4C illustrate the sequence at breakpoints in detail. AT-rich regions were reported to be the hotspots for the insertion of IS*1* (25), which implied the once presence of another IS*1R* on the right end of the deleted fragment.

The deletions of DNA fragments from the chromosome or plasmid have been closely related to a variety of ISs and are not confined to the loss of ARGs. Although these ISs belong to different IS families and their own transposition mechanisms are very different, they show the same characteristics in mediating DNA fragment deletion. The missing fragments are all broken at the position closely next to corresponding ISs. In previous studies (26, 27), it has been deduced that the homologous recombination system of the bacterial host should have been widely involved in the transfer of ARG-bearing composite transposons (i.e., the transposon with the universal structure, IS→ARG→IS). In this study, we realized that the role of various ISs in bacterial genome evolution is not limited to the transmission of ARGs, which may involve the insertion, deletion, inversion (28), and transfer of DNA fragments and even the formation of hybrid plasmids (29–31). According to detailed sequence analysis of the missing fragments and considering the self-transposition property of ISs, homologous recombination should also have played a very important role in these processes.

### Fitness of ARGs.

**FIG 5:**
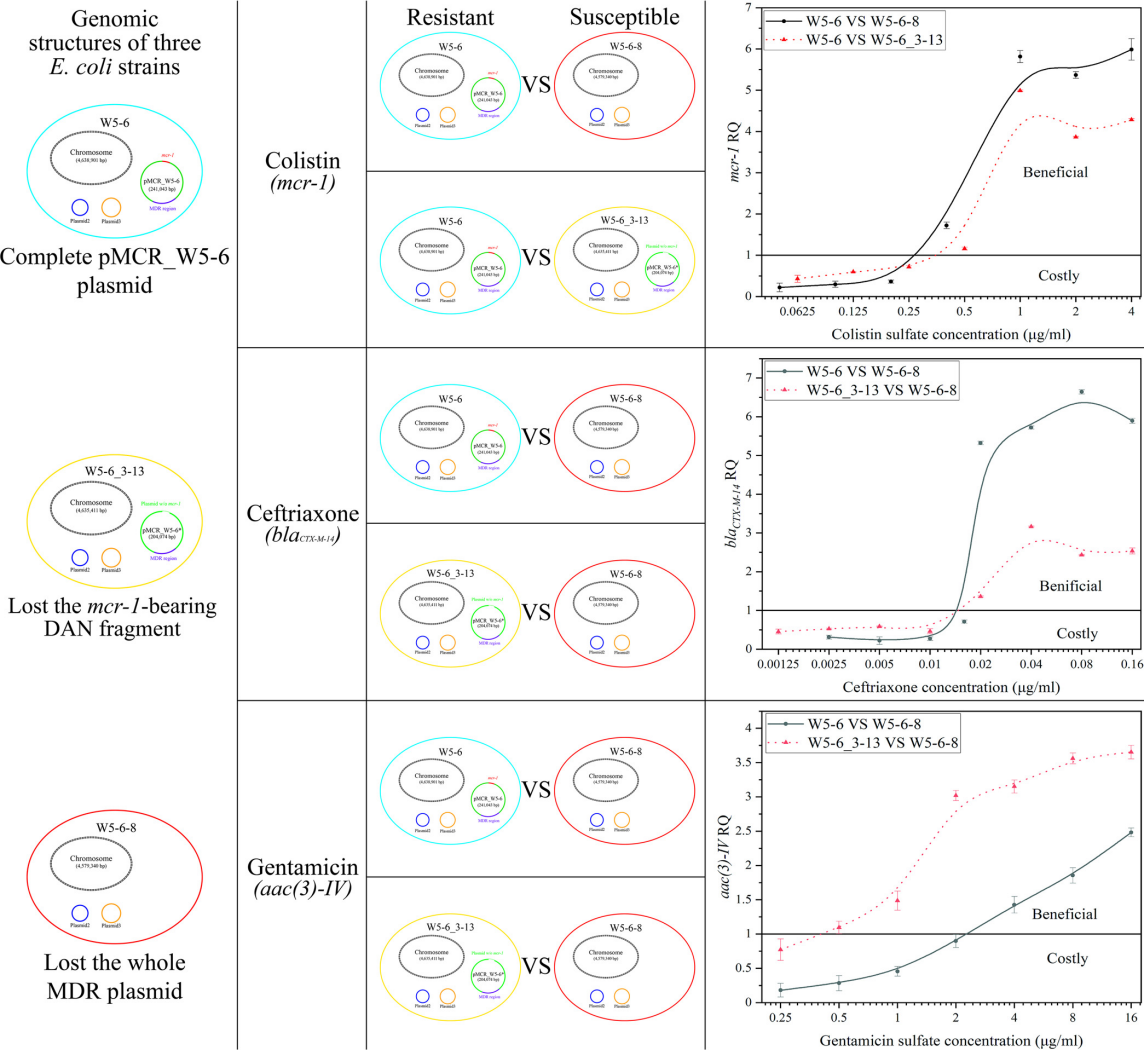
Relative fitness of antibiotic resistance quantified via qPCR. Resistant strain (W5-6 or W5-6_3-13) and susceptible strain (W5-6-8 or W5-6_3-13) are cocultured under the selective pressure of three antibiotics. Relative quantities (RQs) of corresponding ARGs after the coculture were determined via qPCR. The magnitude of RQ relative to 1 reflects the competitive edge of the resistant strain over the susceptible one at different antibiotic concentrations. It also indicates whether the corresponding ARG is costly (RQ < 1) or beneficial (RQ > 1) to the host bacteria at different antibiotic concentrations.

The fitness of antibiotic resistance determinants was evaluated by coculturing the resistant and susceptible E. coli strains at different antibiotic concentrations. As shown in Fig. 5, three sets of experiments were conducted with colistin, ceftriaxone, and gentamicin as selective pressure to evaluate the fitness (relative quantity of target ARG in the target sample in relation to the reference sample [RQ]) of corresponding ARGs [i.e., *mcr-1*, *bla*_CTX-M-14_, and *aac(3)-IV*], respectively. After the 12-h incubation period, all cultures had grown to high turbidity as assessed by eye. The quantification of the 16S rRNA gene also showed that the growth of bacterial cells did not fluctuate too much at different antibiotic concentrations (steady threshold cycle [*C_T_*] values for the 16S rRNA gene; data not shown). Therefore, the low RQ numbers should not be due to lack of growth. First, in the absence of antibiotics or with antibiotics of very low concentration, the RQ values are generally less than 1 after coculture (Fig. 5), which indicates that ARGs (or the MDR plasmid) do endow the drug-resistant bacteria a certain fitness cost, putting them at a disadvantage in competition with susceptible counterparts. This should be the reason why the relative abundances of various ARGs kept decreasing during the successive passage. Second, with increasing the antibiotic concentration, RQ values gradually increased and exceeded 1, indicating that drug-resistant strains gradually occupied the dominant position in the competition.

We intended to use different antibiotics as selective pressure to evaluate the fitness of corresponding ARGs. However, after analyzing the genomic differences between resistant and susceptible strains, we found that this was not accurate. As shown in Fig. 5, for each set of experiments using certain antibiotics as selective pressure, two different combinations of resistant and susceptible strains were adopted in the coculture, respectively. In the case of W5-6 versus W5-6_3-13, the resistant strain has only one more DNA fragment containing the *mcr-1* gene than the susceptible one, while in the other cases, the drug-resistant strain has one more whole MDR plasmid than the susceptible one (W5-6 versus W5-6-8 or W5-6_3-13 versus W5-6-8). For the former situation, it can be considered that the fitness cost of the drug-resistant strain mainly comes from the extra ARG, *mcr-1*. But for the latter one, strictly speaking, the fitness cost of the resistant strain comes from the whole MDR plasmid. By comparing the results of two kind of combinations, one can find that the fitness order of the three strains is W5-6-8 > W5-6_3-13 > W5-6 at no antibiotics. When colistin was used as selective pressure, W5-6 began to show competitive advantage over W5-6_3-13 or W5-6-8 at almost the same concentration of colistin, which indicated little fitness difference between W5-6_3-13 and W5-6-8. In addition, it is worth noting that when gentamicin is used as the selective pressure, compared with W5-6, W5-6_3-13 showed competitive advantage over W5-6-8 at a lower gentamicin concentration. In other words, in the range of gentamicin concentration from 0.5 to 2.0 μg/ml, the fitness order of the three strains is W5-6_3-13 > W5-6-8 > W5-6. W5-6 has only one more DNA fragment containing the *mcr-1* gene than W5-6_3-13 on the MDR plasmid, which endows W5-6 more fitness cost over W5-6_3-13 at no or low antibiotic pressure. These results suggest that the fitness cost of the *mcr-1* gene should dominate the fitness cost of the whole MDR plasmid. It has been reported that the expression of *mcr-1* will lead to a decrease in growth rate, cell viability, and competitiveness (22). During the successive passage, we have already observed that other ARGs can be preserved for a longer time when the *mcr-1* is deleted alone (Fig. 1, condition 3). The IS*Apl1*-mediated deletion of the *mcr-1*-containing fragment can be regarded as a fitness evolution of the resistant bacteria. Recently, a very similar phenomenon has been observed in Gram-positive bacteria (32). Although there is no intentional experimental design, this may be the first time it has been observed that the fitness cost of a plasmid mainly comes from specific resistance genes. In addition, the coselection phenomenon was observed between different ARGs on the same MDR plasmid. As we expected, when using ceftriaxone as selective pressure, the RQ of the *mcr-1* gene increased with increasing concentration of ceftriaxone, following the same trend of that of *bla*_CTX-M-14_ (Fig. S2).

10.1128/mSphere.00356-21.2 FIG S2 Coselection effect between *bla*_CTX-M-14_ and *mcr-1* under the selective pressure of ceftriaxone. The RQ of *mcr-1* increases with increasing the concentration of ceftriaxone, which was consistent with that of *bla*_CTX-M-14_. Download FIG S2, JPG file, 2 MB. Copyright © 2021 Wu et al. 2021 Wu et al. https://creativecommons.org/licenses/by/4.0/ This content is distributed under the terms of the Creative Commons Attribution 4.0 International license.

The 2^−ΔΔ^*^CT^* method used in this work provides a feasible approach for evaluating the relative fitness of resistant and susceptible strains at different antibiotic concentrations without introducing any fluorescent markers (15, 16). When the concentration of an antibiotic is lower than the threshold where the RQ value of the corresponding resistance gene suddenly exceeds 1, it will not enrich the corresponding resistance gene, and it will not show coselection effect on other ARGs with genetic linkage. Whether the environmental abundance of a resistance gene will be reversed depends on the fitness cost it caused to the host bacteria or the bacterial community. For mobile ARGs conferring significant fitness costs, the control on environmental emissions of corresponding antibiotics may be of particular importance, which is expected to effectively reverse the environmental abundances of the ARGs and restore the clinical potency of the antibiotics.

### Conclusions.

The decay of antibiotic resistance during successive passage is mainly caused by the elimination of the whole MDR plasmid and the deletion of individual ARGs, and, in most cases, it is dominated by the elimination of the MDR plasmid. The whole-genome sequencing of representative colistin-susceptible isolates gives definite proof for the elimination of the MDR plasmid and the deletion of the *mcr-1* gene. Furthermore, the deletion of DNA fragments does not only take place on the plasmid and is not confined to ARGs. It also occurs on the chromosome and involves the loss of other functional genes. However, the deletions of these DNA fragments present a common feature, i.e., they are all closely related to certain ISs. The high mobility and high fitness cost of some ARGs may exactly imply their poor stability in host bacteria. Strict control on the use of corresponding antibiotics should reverse the widespread dissemination of these ARGs in bacterial communities. The relative quantification method of qPCR can facilely determine the antibiotic concentration when resistant bacteria gain growth advantage against their susceptible counterpart.

## MATERIALS AND METHODS

### Bacterial isolates.

**FIG 6:**
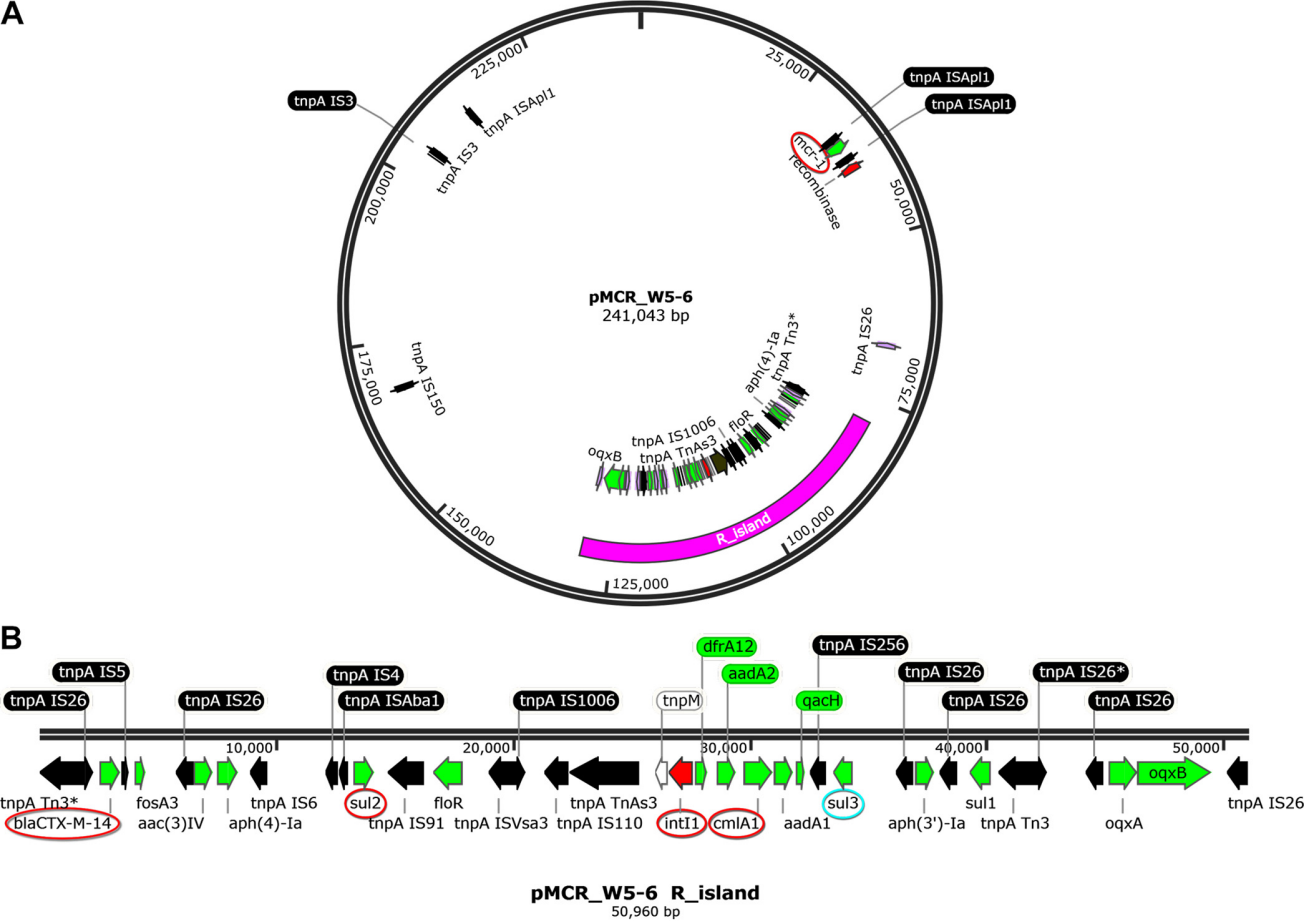
(A) Structure of the multidrug-resistant plasmid (pMCR_W5-6) of E. coli W5-6. (B) Details of the multidrug-resistant region are illustrated. Five typical ARGs and *intI1* (highlighted with colored circles) were chosen for monitoring their stability in the host bacteria.

The *mcr-1*-positive MDR E. coli strain (W5-6) was isolated from an urban river (Jinjiang River, Chengdu, China), and the whole genome was sequenced in our previous study (24). The primary information for the genome of E. coli W5-6 is listed in Table 2. The multidrug resistance of this isolate is mainly conferred by its MDR plasmid pMCR_W5-6 of IncHI2 type. As shown in Fig. 6, the plasmid contains an MDR region where multiple ARGs were assembled with/in a variety of mobile genetic elements, especially various insertion sequences (ISs) and the class 1 integron In640 (accession no. CP032993). The colistin resistance gene *mcr-1* is in another position far away from the MDR region on the plasmid.

### Successive passage experimental setup.

**FIG 7:**
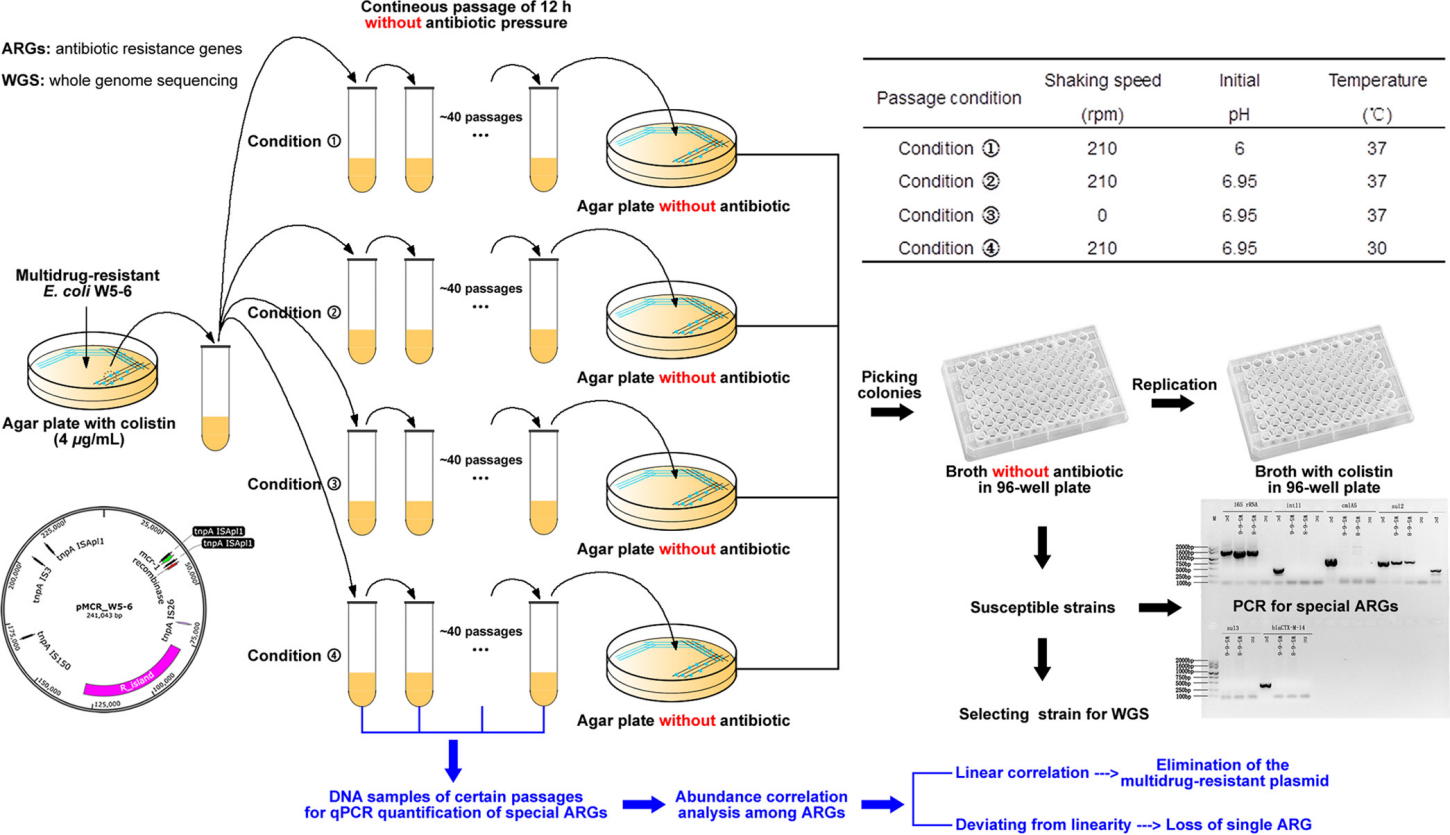
Illustration of the research route. The multidrug-resistant E. coli isolate W5-6, which contains a multidrug-resistant plasmid (pMCR_W5-6), was continuously passaged at four different conditions without antibiotic pressure (refer to the table). At the end of the passage, the susceptible colonies were picked, and the presence of typical ARGs on the multidrug-resistant plasmid was detected. The colonies with specific resistance gene deletion patterns were selected for whole-genome sequencing. DNA samples were extracted from certain passages, and the quantity of special ARGs (ARGs marked with red circles in Fig. 6) was determined via qPCR. Abundance correlation analysis among different ARGs reflected the elimination mode of these resistance determinants.

The research route of this study is illustrated in Fig. 7. E. coli W5-6 was successively passaged under four antibiotic-free culture conditions. The culture conditions are listed in the insertion table of Fig. 7. The possible effects of pH value (condition 1 with initial pH of 6.0), static culture (condition 3 without shaking), and culture temperature (condition 4 with culture temperature of 30°C) on the fitness of antibiotic resistance were considered. Condition 2 was the normal culture condition for E. coli as a control. At each passage, 10 μl of each 12-h culture were inoculated into 5 ml fresh prewarmed medium to provide a suspension of about 10^6^ CFU/ml, which was then incubated for the next 12 h. The number of passages was 40. During the passage, the relative abundances of multiple ARGs and the class 1 integrase gene (*intI1*) were determined via quantitative PCR (qPCR). At the end of the passage, colistin-susceptible strains were isolated from culture of the last passage via colony screening with a 96-well plate. Fifteen colonies were analyzed for their resistance (susceptibility) against colistin for each culture condition, respectively. The presence of specific ARGs in the susceptible isolates was detected with PCR. Two susceptible isolates with special loss mode of ARGs were chosen for whole-genome sequencing.

### qPCR and PCR.

The contents of specific ARGs (*mcr-1*, *cmlA1*, *sul2*, and *bla*_CTX-M-14_) and *intI1* (genes circled in Fig. 6) were followed via qPCR during the successive passage. The amount of each target gene was expressed as a relative abundance versus the 16S rRNA gene (33). All selected target genes, including *intI1*, were on the same MDR plasmid (Fig. 6). The colistin-susceptible strains isolated at the end of the passage were detected for the presence of these specific ARGs and *intI1* via PCR. The primer pairs used in qPCR and PCR are listed in Table S1 in the supplemental material.

10.1128/mSphere.00356-21.3 TABLE S1 Primer pairs for detecting the presence and relative abundance of multiple ARGs. Download Table S1, DOCX file, 0.03 MB. Copyright © 2021 Wu et al. 2021 Wu et al. https://creativecommons.org/licenses/by/4.0/ This content is distributed under the terms of the Creative Commons Attribution 4.0 International license.

### Whole-genome sequencing.

Two colistin-susceptible isolates were subjected to whole-genome sequencing. According to the PCR detection, one isolate should have eliminated the whole MDR plasmid (W5-6-8), and the other one (W5-6_3-13) lost the *mcr-1* gene. Before the whole-genome sequencing, the 16S rRNA gene was amplified for the two isolates and subjected to Sanger sequencing to ensure the purity of the bacterial culture. The genomes of the two isolates were sequenced using single-molecule, real-time (SMRT) technology performed at Beijing Novogene Bioinformatics Technology Co., Ltd. The treatment of sequencing data followed the procedure in our previous study (24).

### Fitness evaluation.

The fitness of antibiotic resistance was evaluated by coculturing resistant and susceptible strains at different antibiotic concentrations. Under the selective pressure of colistin at the concentration ranging from 0.0625 to 4 μg/ml, resistant strain A (W5-6) was cocultured with susceptible strain B (W5-6-8) or C (W5-6_3-13). Under the selective pressure of ceftriaxone at the concentration range of 0.00125 to 0.16 μg/ml, susceptible strain B was cocultured with resistant strain A or C. Under the selective pressure of gentamicin at the concentration range of 0.25 to 16 μg/ml, susceptible strain B was cocultured with resistant strain A or C. The initial mixture of resistant and susceptible E. coli strains was prepared with corresponding pure culture at a cell ratio of resistant strain to susceptible one around 1:2. Part of the initial bacterial mixture was stored frozen for the following DNA extraction. At the same time, 50 μl of the initial mixture were inoculated into 5 ml LB broth with different antibiotic concentrations to provide suspensions of about 10^6^ CFU/ml and were cultured at 37°C overnight (for around 12 hours). Finally, DNA was extracted from all the cultured and initial bacterial mixtures, respectively. The initial bacterial mixture was used as the reference sample to observe the change of relative abundances of ARGs after the overnight culture at different antibiotic concentrations.

For the selective pressure of different antibiotics, relative quantities of corresponding ARGs, *mcr-1* for colistin, *bla*_CTX-M-14_ for ceftriaxone, and *aac(*3*)-IV* for gentamicin, were determined via qPCR according to the 2^−ΔΔ^*^CT^* method (34), which were calculated according to the following equations.

(1) $$\Delta C_{T}=C_{T}\left(\text{target gene}\right)\;-\;C_{T}(\text{reference gene})$$

(2) $$\Delta \Delta C_{T}=\Delta C_{T}\left(\text{target sample}\right)\;-\;\Delta C_{T}(\text{reference sample})$$

(3) $$\text{RQ}=2^{-\Delta \Delta \;\mathrm{CT}}$$

where *C_T_* is the number of cycles experienced when the fluorescence intensity reaches the set threshold, and RQ is the relative quantity of target ARG in the target sample in relation to the reference sample. The 16S rRNA gene was used as the reference gene. The initial mixture of resistant and susceptible E. coli strains was used as the reference sample, and the coculture mixtures at different antibiotic concentrations were the target samples. The expected result is that under no or low antibiotic selective pressure, the resistant strain is at a disadvantage in the competition due to the fitness cost of ARGs, and the RQ value should be less than 1. On the contrary, under high selective pressure, susceptible bacteria will be inhibited, and RQ value should be greater than 1. Thus, the value of RQ reflects the relative fitness of the resistant strain against susceptible one under different antibiotic concentrations.

### Statistical analysis.

In order to assay the synchronicity of ARGs (including *intI1*) on the same MDR plasmid during the successive passage, Pearson correlation coefficients between relative abundances of different ARGs were calculated, and the significance was evaluated with SPSS.
