# Supplementary figures and images for: Reversing Antibiotic Resistance Caused by Mobile Resistance Genes of High Fitness Cost
Source: mSphere. 2021 Jun 23;6(3):e00356-21. doi: 10.1128/mSphere.00356-21 (PMC8265650; doi:10.1128/mSphere.00356-21)

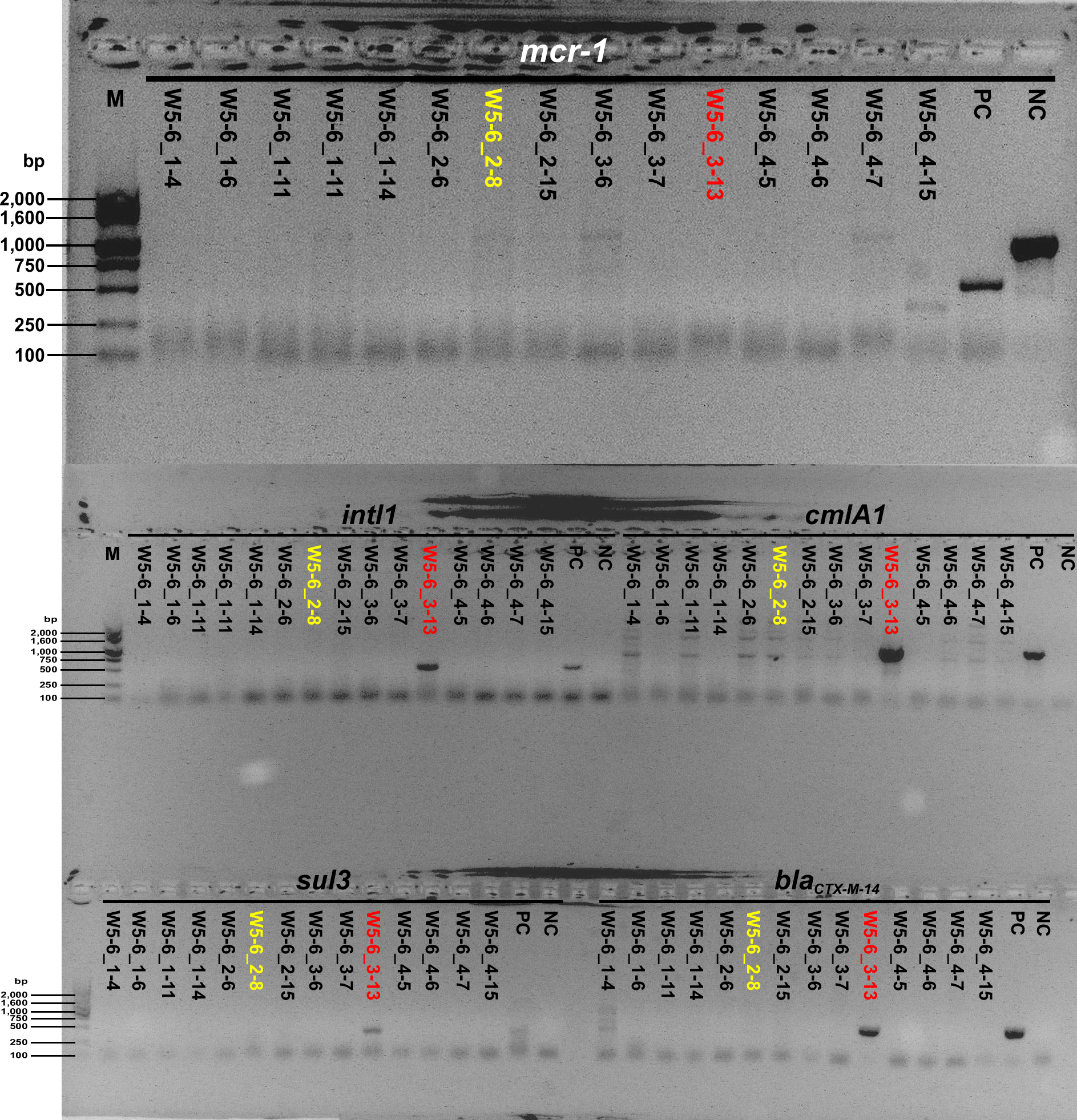

Supplement: FIG S1 [file msphere.00356-21-sf001.jpg]

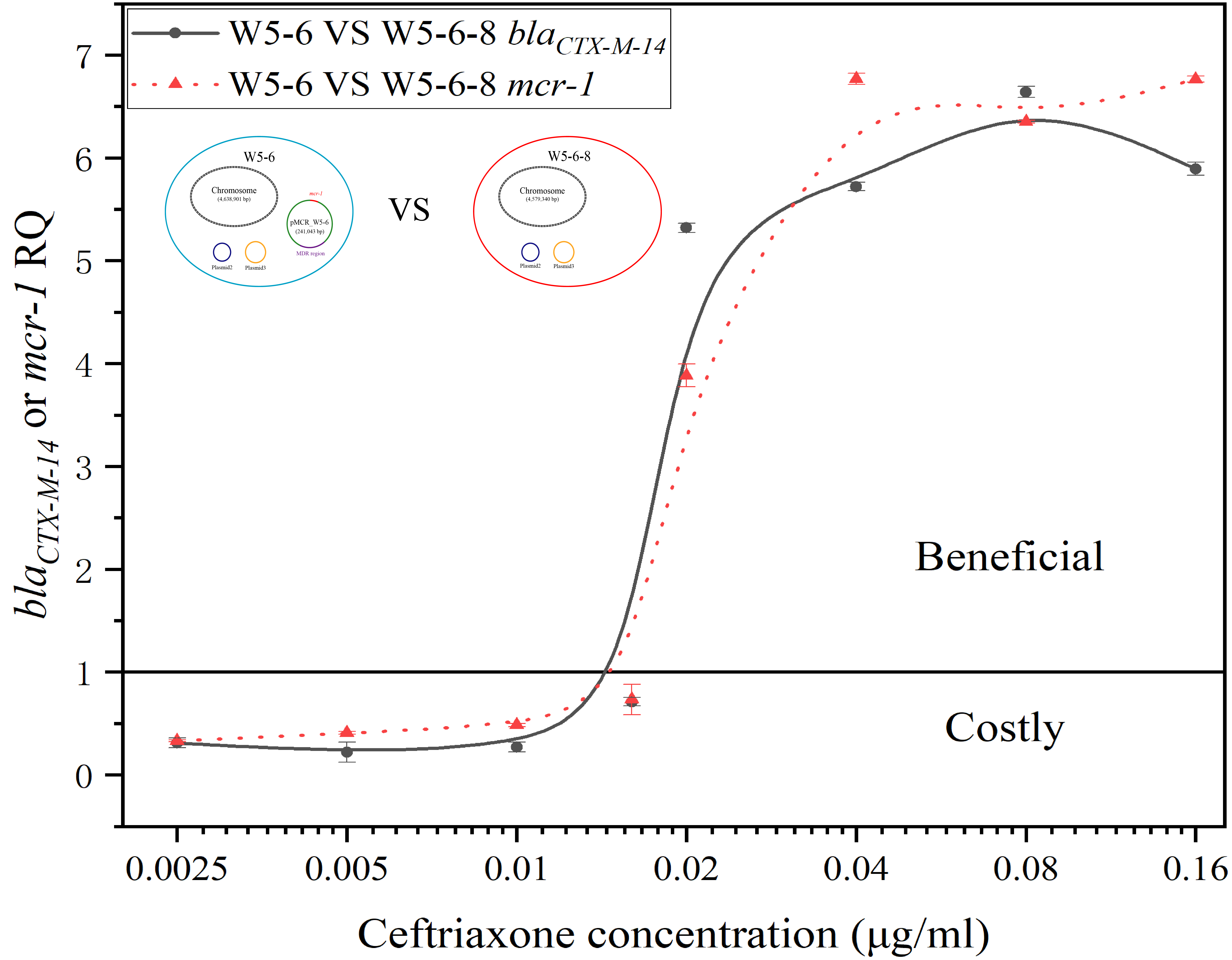

Supplement: FIG S2 [file msphere.00356-21-sf002.jpg]
