# Supplementary material for: Reversing Antibiotic Resistance Caused by Mobile Resistance Genes of High Fitness Cost
Source: mSphere. 2021 Jun 23;6(3):e00356-21. doi: 10.1128/mSphere.00356-21 (PMC8265650; doi:10.1128/mSphere.00356-21)
Supplement: TABLE S1 [file msphere.00356-21-st001.docx]

**Table S1. Primer pairs for detecting the presence and relative abundance of multiple ARGs**

| Target gene | Gene function | Primer For（5’→3’） | Primer Rev.（5’→3’） | Product size（bp） | Annealing temperature (℃) |
| --- | --- | --- | --- | --- | --- |
| 16S rDNA | 16S rRNA | 27F: AGAGTTTGATCMTGGCTCAG | 1522R: AAGGAGGTGATCCANCCRCA | 1542**^a^** | 59 |
| 16S rDNA | 16S rRNA | GGGTTGCGCTCGTTGC | ATGGYTGTCGTCAGCTCGTG | 60**^b^** | 59 |
| *intl1* | Class 1 integrase | GGGTCAAGGATCTGGATTTCG | ACATGCGTGTAAATCATCGTC | 484 | 62 |
| *sul2* | Resistance to sulfonamides | CATCATTTTCGGCATCGTC | TCTTGCGGTTTCTTTCAGC | 793 | 62.5 |
| *sul3* | Resistance to sulfonamides | TAGATGTTTCTGGATTAGAGCCT | AGATGTGATTGATTTGGGAGC | 443 | 62.5 |
| *cmlA1* | Resistance to chloramphenicol | TGCCAGCAGTGCCGTTTAT | CACCGCCCAAGCAGAAGTA | 957 | 62 |
| *bla*_CTX-M-14_ | Resistance to *β*-lactam | GCTGGAGAAAAGCAGCGGAG | GTAAGCTGACGCAACGTCTG | 474 | 66.5 |
| *mcr-1* | Resistance to colistin | CGGTCAGTCCGTTTGTTC | CTTGGTCGGTCTGTAGGG | 309 | 59 |

^a^: The primer pair target 16S rDNA with long product (1542 bp) was only used in PCR and Sanger sequencing to prove the bacterial strain was still *E. coli* during the successive passage without contamination (1).

^b^: The primer pair target 16S rDNA with short product (60 bp) was used in qPCR to quantify the amount of *E. coli* in the culture (2).

Note: All other primer pairs were obtained from references (3-6) and used both in PCR and qPCR. The qPCR adopted the three-step mode, i.e., denaturing at 95 ^o^C for 20 s, annealing at certain temperature for 20 s, extending at 72 ^o^C for 30 s, and keeping the thermal cycle for 42 rounds.

**Supplementary references**

1. Buchan A, Newell SY, Butler M, Biers EJ, Hollibaugh JT, Moran MA. 2003. Dynamics of bacterial and fungal communities on decaying salt marsh grass. Appl Environ Microbiol 69:6676-87.

2. Zhu YG, Johnson TA, Su JQ, Qiao M, Guo GX, Stedtfeld RD, Hashsham SA, Tiedje JM. 2013. Diverse and abundant antibiotic resistance genes in Chinese swine farms. Proc Natl Acad Sci U S A 110:3435-40.

3. Zhang AY, Wang HN, Tian GB, Zhang Y, Yang X, Xia QQ, Tang JN, Zou LK. 2009. Phenotypic and genotypic characterisation of antimicrobial resistance in faecal bacteria from 30 Giant pandas. Int J Antimicrob Agents 33:456-60.

4. Pitout JD, Hossain A, Hanson ND. 2004. Phenotypic and molecular detection of CTX-M-beta-lactamases produced by Escherichia coli and Klebsiella spp. J Clin Microbiol 42:5715-21.

5. Saenz Y, Brinas L, Dominguez E, Ruiz J, Zarazaga M, Vila J, Torres C. 2004. Mechanisms of resistance in multiple-antibiotic-resistant Escherichia coli strains of human, animal, and food origins. Antimicrob Agents Chemother 48:3996-4001.

6. Liu YY, Wang Y, Walsh TR, Yi LX, Zhang R, Spencer J, Doi Y, Tian G, Dong B, Huang X, Yu LF, Gu D, Ren H, Chen X, Lv L, He D, Zhou H, Liang Z, Liu JH, Shen J. 2016. Emergence of plasmid-mediated colistin resistance mechanism MCR-1 in animals and human beings in China: a microbiological and molecular biological study. Lancet Infect Dis 16:161-8.
